# Effect of Post-Weld Heat Treatment on Microstructure and Hardness of Laser Beam Welded 17-4 PH Stainless Steel

**DOI:** 10.3390/ma16041334

**Published:** 2023-02-04

**Authors:** Lechosław Tuz, Łukasz Sokołowski, Sebastian Stano

**Affiliations:** 1 Faculty of Materials Engineering and Industrial Computer Science, AGH University of Science and Technology, 30-059 Krakow, Poland; 2 Łukasiewicz Research Network—Institute of Welding, 44-100 Gliwice, Poland

**Keywords:** laser, beam, welding, alloy, steel, welding, hardness, microstructure, heat, treatment

## Abstract

This article presents the results of research on the development of the technology of laser beam butt welding of 17-4 PH stainless steel sheets and the technology of post-weld heat treatment (PWHT). The developed technology allows for favorable conditions to be obtained and for the appropriate microstructure and hardness to exist in the weld area. Moreover, it enables the fulfillment of a number of specific requirements beyond the possibilities of manual welding and other methods. The tests performed include the analysis of the microstructure with the use of light microscopy (LM) for the materials after welding and PWHT. The applied PWHT showed changes in the microstructure and mechanical properties. In all weld areas the martensitic microstructure was observed. The homogeneity of the microstructure in the area of the welded joint after PWTH was revealed. In the as-welded condition and after the PWHT with aging at 481 °C, the hardness was 440 HV5, but after aging at 621 °C, it decreased to 330–340 HV5.

## 1. Introduction

The development of industry means that the materials used are required to work in difficult conditions, with very good corrosion resistance and high strength. Due to the combination of these properties, 17-4 PH steel is used successfully in the aviation [1], energy [2], shipbuilding [3], and petrochemical industries [4,5,6,7]. Its good corrosion resistance is an effect of the chemical composition and its high strength is reached in a two-stage heat treatment [8,9].

The 17-4 PH stainless steel (X5CrNiCuNb16-4) is a corrosion resistant steel with a martensitic structure [1,9], good weldability [6], and wear resistance [7]. Obtaining a martensitic structure and the required mechanical properties requires a heat treatment that involves supersaturation at a temperature of approximately 1020–1050 °C while cooling in the air and then aging at a temperature in the range of 480–620 °C [2]. Aging ensures the release of the dispersion Cu precipitates that are coherent with the matrix and constitute the strengthening phase, where too long an aging time causes the growth of copper precipitates and, at the same time, a decrease in hardness (mechanical properties) [4]. The low carbon content makes it possible to limit the formation of chromium carbides, ensuring the high ductility of martensite. Supersaturation at 1020–1050 °C ensures the dissolution of precipitates in a solid solution, where the copper precipitates (ε phase) dissolve already at 920 °C, and the chromium-rich phases (σ, Z, M_23_C_6_) dissolve above 950 °C. Then, precipitates of niobium carbides may remain mainly undissolved [10,11,12]. This indicates that when the 17-4 PH steel components are welded, the strengthening phase will dissolve in the high temperature heat affected zone (HAZ) [8,13]. In addition, the use of a filler material (FM) with a chemical composition compatible with the base material (BM) will result in the weld being non-hardening and therefore, it will show lower mechanical properties than the BM in the condition after the heat treatment (PWHT). This requires the post-welding heat treatment of the welded joints.

The good weldability of 17-4 PH steel means that conventional arc welding processes are now used for welding, such as gas tungsten arc welding (GTAW) and shielded metal arc welding (SMAW) [14,15]. These processes make it possible to join both thin and thick sheets [15]. During welding, however, it is necessary to properly bevel the edges and, as a result, add filler material. On the other hand, the heat input in arc welding causes overaging of the steel, and consequently, it is necessary to reheat them, which can lead to deformation in the products. Moreover, with significant thicknesses, preheating is also often used to reduce the susceptibility to cracking [4,11].

To make high-quality joints of materials without decreasing the strength properties, conventional methods are increasingly being replaced by laser welding. Welding with a laser beam makes the joints obtained characterized by high quality and accuracy, which is the effect of a high power density and low heat input. Low heat input is a consequence of a high welding speed. Such process parameters allow it to reach a deep penetration and a narrow heat affected zone (HAZ), but they also can strongly influence the porosity or cracks which is very challenging [15]. The analyses conducted on the impact of the heat input during welding cover a number of different issues related to the shape of the joint, e.g., circumferential welds [16] or after repair [17], or joining thin or thick materials [18,19,20]. Hybrid processes [20,21], as well as a specific type of generated beam [22,23,24,25] are also gaining importance. A separate guess is the issue of the impact of the heat input into the material and post-weld heat treatment of both 17-4 PH steel and other stainless steels, e.g., austenitic [19,23,26,27], ferritic [24], duplex [28], or aluminum [29,30]. This indicates that despite the fact that construction materials are characterized by good weldability, the method and effect of the heating and cooling, as well as the possible subsequent heat treatment [31] are of key importance for mechanical, plastic, or operational properties [12,13,32].

This work contains the results of microstructure and hardness tests obtained in the newly developed technology of laser welding of 17-4 PH steel in the state after welding and after a two-stage heat treatment (PWHT). The use of available laser devices was of key importance for the technology being developed, while the results of technological trials were not discussed in the paper. The joint made with favorable welding parameters was subjected to detailed tests due to the microstructure and mechanical properties. The results obtained for the as-welded conditions were related to the microstructure and properties obtained after the heat treatment, including supersaturation and aging.

## 2. Materials and Methods

**Figure 1:**
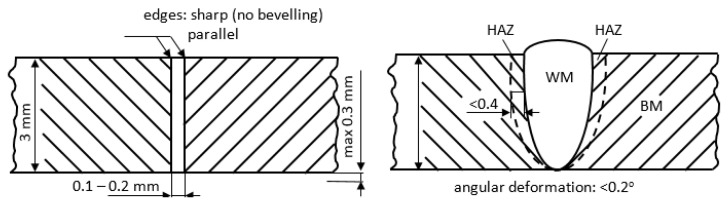
The joint design requirements.

The research included developing a technology for welding butt joints with the use of laser beam welding (LBW) and then carrying out the PWHT to obtain the required mechanical properties and microstructure. The materials for the tests were sheets made of precipitation-hardened stainless steel 17-4 PH—X5CrNiCuNb16-4; 1.4548 (250 mm × 100 mm × 3 mm). Welding was performed using a disc laser of 521 acc. to EN 4063 without filler metal (FM). Figure 1 shows the requirements for preparing edges for welding and the required weld dimension. In Table 1 the LBW parameters are presented.

The chemical composition analysis was performed by optical emission spectroscopy (OES) using a Foundry Master WAS spectrometer (Table 2).

The quality assessment of the welded joints was carried out on the basis of the non-destructive testing (NDT) method, including visual testing (VT) and liquid penetrant inspection (PT). In VT the requirements of EN ISO 17637 and EN 13018 were met. The VT was carried out by a direct method in intense lighting of about 760 lx with a universal welding gauge with a resolution of 0.01 mm. The PT inspection met the ISO 3452-1 requirements with the use of a colorful penetrant with kit II C e-2 and a light intensity of over 500 lx. The penetration and development time was 30 min, respectively. For the NDT test, the acceptance level was B (highest) acc. To 13919-1 for the VT. The post-weld heat treatment (PWHT) was carried out with the use of a FCP 3.5 chamber furnace equipped with a SM 946 temperature controller. The PWHT parameters are presented in Table 3.

Macro- and microscopic examinations were carried out in the cross-section after mechanical grinding, polishing, and electrochemical etching in a 10% CrO_3_ + 90% H_2_O solution for about 10 s. The microstructure was characterized using the light microscopes (LM) Leica Stereozoom S9i (Leica, Wetzlar, Germany) and Leica DM/LM (Leica, Wetzlar, Germany).

Hardness measurements were made using the Vickers method with an intender load of 10 kG (98.07 N), using a Zwick/Roell ZHU 187.5 hardness tester (Zwick Roell Group, Ulm, Germany).

## 3. Results and Discussion

### 3.1. NDT Tests

**Figure 2:**
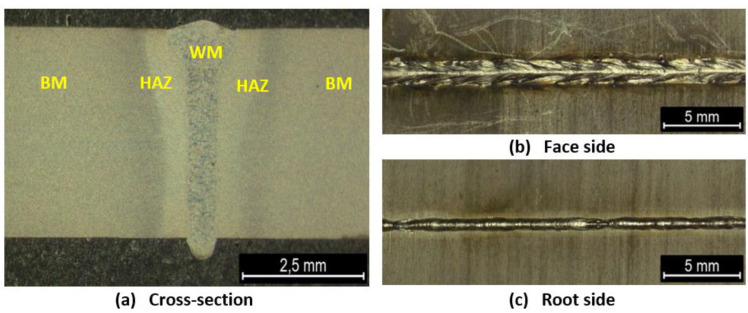
The macrostructure of the joints in the cross-section (**a**) and on the face (**b**) and root (**c**) side; WM—weld metal, HAZ—heat affected zone, BM—base metal.

The VT revealed the correct shape of the face that meets the requirements of quality level B acc. to ISO 13919-1. The VT and PT tests did not reveal cracks or discontinuities, which indicates that the surfaces of the welded joints made did not show incompatibility. The face was characterized by high regularity. The root showed the correct shape and uniform width throughout its entire length (Figure 2).

### 3.2. Macroscopic Examination

The macrostructure of the sample cross-section (Figure 2a) showed the symmetrical nature of the WM. Its shape and width were uniform throughout the joint length. The face (Figure 2b) had a convex shape with a smooth transition into the BM, while the root (Figure 2c) showed a large relief with a sharp transition into the BM. The cross-section showed that the heat affected zone (HAZ) was characterized by two areas. The HAZ areas were of the same width on both sides of the joint. The observation of the joint cross-section did not reveal any visible subsurface defects.

### 3.3. Microscopic Examination

**Figure 3:**
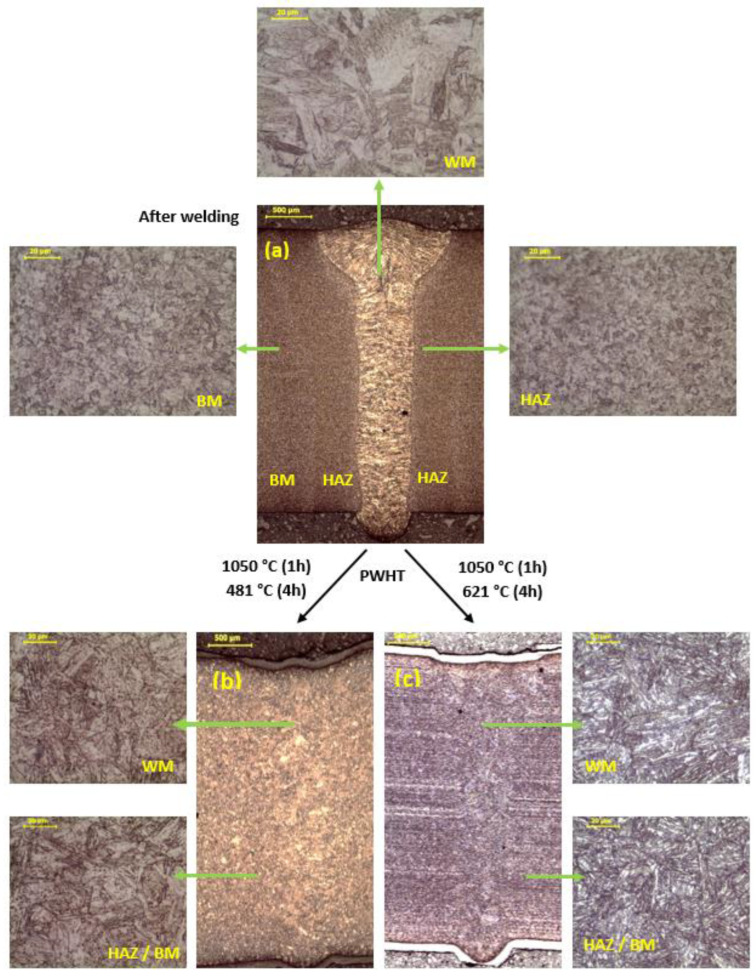
The microstructure of the joint after: (**a**) welding, (**b**) PWHT (1050 °C/481 °C), and (**c**) PWHT (1050 °C/621 °C).

The microscopic examination (Figure 3a) revealed the presence of a coarse-grain martensitic structure in the WM. Crystallites grew from the fusion line, both on the right and left sides, aligning with the axis of the weld towards the face. The structure in both regions of the HAZ showed a fine crystalline martensitic character. The comparison of these areas shows that the number of secretions that appeared in both areas is the same. In the first HAZ area, the grain boundaries were less visible due to the high temperature that occurs in this area during welding (supersaturation area). In the second region, the temperature was within the aging temperature range. As a result, precipitation processes occurred, which resulted in less visibility of the grain boundaries in the microstructure of second area of the HAZ. The structure of the HAZ was characterized by a structure identical to that of the BM.

In the cross-section of the joint (Figure 3b) subjected to the supersaturation process at 1050 °C for 1 h and the aging process at 481 °C for 4 h, the WM was hardly visible. This proves that as a result of the PWHT the process of homogenization of the entire welding area took place. The grain boundaries of the former austenite were revealed. An observation of the WM revealed grains as large as 20 µm. This grain size classified the structure as fine. The microstructure of the BM showed that it does not differ structurally from that of the WM. The difference appeared in the size of the grains. The grains found in the BM were characterized by smaller sizes than those of the WM.

In the cross-section of the joint (Figure 3c) subjected to the process of supersaturation at the temperature of 1050 °C for 1 h and the aging process at the temperature of 621 °C for 4 h, the weld was practically invisible (uniformity of the WM). In the welding area, the banding of the structure, created by the rolling of the sheet, which was visible in the BM, was lost. The observation of the microstructure of the WM showed a martensitic structure with sharp visible grain boundaries of the former austenite. The structure of the WM differed in morphology from that of the BM.

### 3.4. Hardness Testing

**Figure 4:**
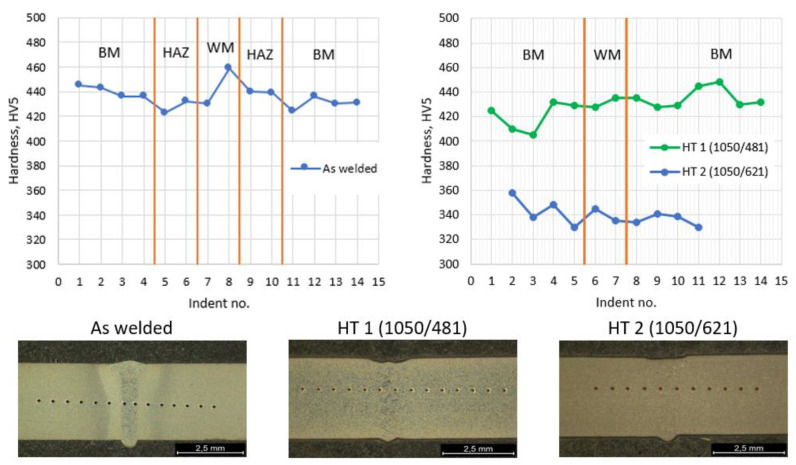
The Vickers hardness distribution in the cross-section for the conditions of the as-welded and after heat treatment with the indent placement.

The distribution of the hardness after welding was even in all areas of the welded joint. The hardness ranged from 425 HV5 to 460 HV5. The HV5 hardness distribution (Figure 4) shows a lack of significant changes for the PWHT with aging at 481 °C (approximately 440 HV5) and a decrease in hardness to about 330–340 HV5 after aging at 621 °C compared to the as-welded conditions (440–450 HV5). It showed that the applied PWHT (1050/621) strongly affected the mechanical properties of the joint. Both in the joints after as-welding and after the PWHT, no significant differences in hardness between the HAZ and BM were observed. Since in the samples after the PWHT, the HAZ area was macroscopically invisible as an effect of the structure homogenization, the comparison of the hardness was related to the analogous areas revealed for the as-welded condition.

### 3.5. Discussion

In order to assess the impact of the heat treatment on the microstructure and hardness of welded joints made with a laser beam, a welded joint meeting the technological requirements was made. The high quality of the joint was confirmed in NDT tests. The welded joints subjected to NDT tests revealed compliance with the technological requirements and met the requirements of the B quality level according to EN ISO 13919 (Figure 1 and Figure 2). The joint was characterized by full penetration and a narrow HAZ (Figure 2a) which the macroscopic examination shows. No cracks were found in the NDT tests. This indicates that the use of a laser beam for welding 17-4 PH steel with the thickness up to 3 mm is beneficial in terms of the shape of the welded joint. The aim of the technological trials was to develop a laser beam welding technology that would provide a favorable amount of heat input into the material, ensuring an even distribution of hardness in the state after welding, which could be the basis for resigning from the PWHT. The tests performed to assess the effect of the PWHT on the microstructure revealed that as a result of supersaturation, the microstructure was homogenized in the welded joint and caused the coarse-grained structure of the WM to disappear. The weld area (WM) after welding had a coarse-grained microstructure typical of casting processes (Figure 3a).

Due to the thermal conductivity of steel, the heat is dissipated to adjacent areas (HAZ), causing structural changes or initiating precipitation processes [8,9]. This means that by limiting the amount of heat input into the material at the appropriate welding speed, it is possible to make high-quality joints with favorable mechanical properties without causing significant (adverse) structural or mechanical changes, which is typical of overaging observed by GTAW or SMAW welding [14,15].

In evaluating the structural and mechanical properties in the as-welded condition, the heat treatment (PWHT) was performed at 481 °C and 621 °C (Table 3). The test results showed that both in the as-welded condition and after the PWTH, a martensitic structure typical of 17-4 PH stainless steel was observed (Figure 3). On the other hand, comparing the hardness distributions in the as-welded condition and the PWTH condition at 481 °C, it can be observed that the hardness (HV5) was similar both in all areas of the joint and between the as-welded condition and the PWTH (Figure 4). The obtained hardness values were approx. 440 HV5. This indicates that the copper strengthening effect was taking place. The strengthening phase consists of finely dispersed copper precipitates coherent with the matrix [1,2,8]. Therefore, the high welding speed (short welding time) did not cause coagulation of the copper precipitates that could lead to a decrease in the hardness. The PWHT at 621 °C caused the reinforcing phase to become incoherent with the matrix, which in turn led to a decrease in the hardness to approximately 330–340 HV5 [4].

## 4. Conclusions

The VT showed that the tested joint met the quality level B according to PN-EN ISO 13919. This shows that it is possible to make a correct joint welded with a LBW of 17-4 PH stainless steel with thickness of 3 mm.

The joint was characterized by a slightly convex face and correct root fusion. Macroscopic examination revealed a symmetrical shape of the weld with a coarse-grained structure in two areas of the heat-affected zone, which are structurally identical. Microscopic examination showed that the sample had the typical martensitic structure of 17-4 PH steel joints. The PWHT led to homogeneity of the weld area and the base material. Both the as-welded and after PWHT condition did not reveal the hardening phase precipitation visibility during microstructural observations.

In the as-welded condition, the hardness was similar in each area of the joint and ranged between 425 and 460 HV5. The lack of significant changes in the HAZ indicates that the amount of heat input into the material did not cause significant changes in the HAZ that could result in the loss of (decrease) mechanical properties.

An even distribution of hardness was observed in both the as-welded and PWHT conditions. The applied aging temperature of 481 °C provided a hardness similar to that of the as-welded condition of approximately 440 HV5, which indicated that both after welding and after PWHT, the precipitation of the strengthening phase remained coherent with the matrix. In the case of the sample subjected to aging at a temperature of 621 °C, the hardness decreased to 330–340 HV5, which could have been due to the effect of copper precipitates coagulation. Coarse precipitations are incoherent with the matrix which decreases the hardening effect.

This developed technology provides the possibility of obtaining favorable mechanical properties after welding and heat treatments. In low-temperature operating conditions, the joints can be operated in the post-weld condition, where a martensitic structure is obtained during welding, but for applications requiring higher loads, PWHT is necessary and obligatory for the homogenization of the microstructure.

## Figures and Tables

**Table 1:** The LBW parameters used for welding.

Parameter	Value
Beam pattern	CW mode
Welding speed	2000 mm/min (33.3 mm/s)
Focal length	300 mm
Laser power	2000 W
Beam diameter	0.4 mm
Heat input	60 J/mm

**Table 2:** The chemical composition of 17-4 PH stainless steel used for welding.

Element		C	Si	Mn	S	P	Cr	Ni	Cu	Nb+Ta
**EN 10088-1**	**Min.**	-	-	-	-	-	15.00	3.00	3.00	0.15
	**Max.**	0.07	1.00	1.00	0.03	0.04	17.50	5.00	5.00	0.45
**OES**		0.06	0.69	0.84	0.02	0.02	16.23	3.69	3.42	0.24

**Table 3:** The PWHT parameters.

Post Weld Heat Treatment						
Supersaturation				Aging		
Sample	T	*t*	Cooling	T	*t*	Cooling
B	1070 °C	1 h	air *	481 °C	4 h	air *
C	1070 °C	1 h	air *	621 °C	4 h	air *

* Required: less than 1 h to 25 °C.

## Data Availability

All data are provided in full in the results section of this paper.
